# Draft Genome Sequence of *Clostridium sporogenes* Strain UC9000 Isolated from Raw Milk

**DOI:** 10.1128/genomeA.00244-16

**Published:** 2016-04-14

**Authors:** Angela La Torre, Daniela Bassi, Teresa Zotta, Luigi Orrù, Antonella Lamontanara, Pier Sandro Cocconcelli

**Affiliations:** a Istituto di Microbiologia, Università Cattolica del Sacro Cuore, Cremona, Italy; b Istituto di Scienze dell’Alimentazione, CNR, Avellino, Italy; c Consiglio per la ricerca e la sperimentazione in agricoltura e l’analisi dell’ economia agraria, Centro di ricerca per la genomica vegetale (CREA-GPG), Fiorenzuola d’Arda, Italy

## Abstract

*Clostridium sporogenes* is a causative agent of food spoilage and is often used as the nontoxigenic surrogate for *Clostridium botulinum*. Here, we described the draft genome sequence and annotation of *C. sporogenes* strain UC9000 isolated from raw milk.

## GENOME ANNOUNCEMENT

*Clostridium sporogenes* represents a good microbiological model for the study of Gram-positive, spore-forming, anaerobic bacteria because it can cause food spoilage (1), it occasionally appears as a clinical pathogen (2), and it shares nearly identical metabolic properties with *Clostridium botulinum* Group I (3, 4), except for in the area of toxin production. For these reasons, it has often been used as a surrogate of *C. botulinum* in demonstrating the effectiveness of food preservation processes (4, 5) and in germination studies (6, 7). Although the analysis based on 16S rRNA sequence comparison (8, 9) grouped the two species together in a single clade, and Weingand et al. (10), on the basis of the core genome analysis, proposed that *C. sporogenes* and *C. botulinum* formed two related but separated clades, further genomic data are needed to better understand the genetic relationships between these two species and their classification in taxonomic units.

In the current work, we propose a *de novo* shotgun sequencing of *C. sporogenes* strain UC9000 isolated from raw milk and responsible of hard cheese spoilage. The genome was sequenced with a 700-fold overall genome coverage using an Illumina HiSeq 1000 platform from IGA Technology Services (Udine, Italy). The reads set were *de novo* assembled using the CLC Genomic Workbench software (version 8.0.3). This strategy resulted in 111 contigs with a calculated genome size of 4.3 Mb and a G+C content of 27.8%. A total of 4,151 genes were predicted by annotating the genome with both the NCBI Prokaryotic Genome Automatic Annotation Pipeline (PGAP) and the RAST Annotation Server (11, 12); 3,932 of the genes are coding sequences (CDS), and there are151 pseudogenes, 1 rRNA, and 62 tRNAs.

The proteolytic nature of this strain was reflected in the genome by the presence of genes involved in amino acid metabolism. The strain also harbors genes involved in sugar metabolism, transport, and uptake, like genes participating in chitin and *N*-acetylglucosamine utilization. Genes coding for botulism neurotoxins and accessory nontoxin-nonhemaglutinin (NTNH) were not detected. We also identified genes involved in DNA metabolism (coding for clustered regularly interspaced short palindromic repeat [CRISPR]-associated proteins and restriction-modification systems), in spore germination (*ger*AA, *ger*AB, *ger*AC, *cwl*J and *sle*B homologues), in motility and chemotaxis. Finally, two intact and three partial phage regions were identified by using the PHAge Search Tool (PHAST) (13), while transposable elements belonging to IS200/IS605 and IS66 families were retrieved from the IGS Annotation Engine and Manatee web-based tool (14).

### Nucleotide sequence accession numbers.

This complete genome sequence has been deposited at DDBJ/EMBL/GenBank under the accession no. LJFK00000000. The version described in this paper is version LJFK01000000.
